# Cormorant Catch Concerns for Fishers: Estimating the Size-Selectivity of a Piscivorous Bird

**DOI:** 10.1371/journal.pone.0077518

**Published:** 2013-11-07

**Authors:** Vladimir Troynikov, Athol Whitten, Harry Gorfine, Žilvinas Pūtys, Eglė Jakubavičiūtė, Linas Ložys, Justas Dainys

**Affiliations:** 1 Department of Zoology, University of Melbourne, Melbourne, Victoria, Australia; 2 Laboratory of Marine Ecology, Nature Research Centre, Vilnius, Lithuania; North Carolina State University, United States of America

## Abstract

Conflict arises in fisheries worldwide when piscivorous birds target fish species of commercial value. This paper presents a method for estimating size selectivity functions for piscivores and uses it to compare predation selectivities of Great Cormorants (*Phalacrocorax carbo sinensis* L. 1758) with that of gill-net fishing on a European perch (*Perca fluviatilis* L. 1758) population in the Curonian Lagoon, Lithuania. Fishers often regard cormorants as an unwanted “satellite species”, but the degree of direct competition and overlap in size-specific selectivity between fishers and cormorants is unknown. This study showed negligible overlap in selectivity between Great Cormorants and legal-sized commercial nets. The selectivity estimation method has general application potential for use in conjunction with population dynamics models to assess fish population responses to size-selective fishing from a wide range of piscivorous predators.

## Introduction

Ecological interactions between cormorants, fish, and fishers are problematic globally [1]–[7]. Among those studies describing the effects of increasing cormorant numbers on fish populations and fisheries in Europe [5], [8] and North America [9]–[11] many suggest cormorants have a negative impact, and that is certainly a common perception of fishers [7]. Other studies have demonstrated that cormorant impact can be negligible [12]–[14], and may not lead to declines of commercial and non-commercial fish species. Cormorants frequently target commercially and recreationally important fish [7], [15], [16] and increases in the size of piscivorous bird colonies can obviously place pressure on fish stocks. A key requirement to understanding the effects of cormorant populations on fish stocks is the size-specific selectivity of their predation, however estimating parameters to describe predation selectivity can be difficult.

Previous studies of cormorant predation have relied predominantly on qualitative and empirical assessments of predation selectivity and population level effects [17]. A number of studies identified length distributions for all prey fish (e.g. [18]–[21]), whereas others focused on the most important species in the diet (e.g. [7], [8], [22]–[24]). Several assessed those fish age groups most affected by cormorant predation (see [25], [26]) or considered seasonal differences in selectivity [27], [28], and one focussed on size and species selectivity effects on fish community structure [29].

Most studies on competition between cormorants and fisheries have concentrated on fish consumption rather than catch composition and size structure (e.g. [8], [13], [19], [20], [30]). Despite some research estimating potential or existing direct competition, to our knowledge, evaluation of the quantitative overlap of cormorant diet and fisheries has not been undertaken previously. Among dietary studies it has been found that cormorants exploit fish mostly smaller than fishery minimum sizes [31]. In a study comparing cormorant diets with anglers' catches in the Great Lakes USA, the authors concluded that each group captured different sizes of fish with only a minor overlap [32]. A similar study in Lithuania compared the size distribution of regurgitated fish with commercial gillnet catches and concluded that direct competition was low [7]. Examining the potential for direct competition with the commercial fishery in Lithuania's Curonian Lagoon, it was found that although legal-sized fish comprised more than one quarter of the cormorants' diet, the composition was dominated by species of low commercial value [24].

Conventional methods for directly estimating selectivity parameters from length-composition data restrict the estimation process to a single type of fishing gear: They are based on the assumption that data are collected by gear types with equal or comparable units of fishing effort (e.g. [33]). Integrated analysis methods allow selectivity parameters for multiple types of gear, and therefore, for multiple functional forms, to be estimated [34]. Such methods combine several sources of data into a single analysis, so that parameters to describe all or most components of a population and fishery can be estimated as part of a single likelihood. Conversely, direct methods of estimating population and fishery related parameters lead to a two-step approach: Data are analysed in their raw form to produce parameter estimates and those estimates are used in population or stock assessment models [35]. The latter approach, whilst being simpler and less computationally and data intense, also allows for selectivity parameters to be estimated as independent properties of fishing gear without statistical correlation with other parameters in a population model.

In this paper we describe methods to directly estimate the parameters of selectivity functions using length composition data; simultaneous estimation of selectivity parameters for both fishing nets and piscivorous predators is achieved without an assumption of equality in fishing power among nets or predators. The methods also allow the estimation of parameters for multiple functional forms of selectivity and provide a basis for incorporating predation selectivity into analyses of cohort dynamics or general size-age-structured models. Such models could in turn be used to determine the effects of piscivores on fishable stocks. This is essential for developing an understanding of actual, compared with perceived, levels of competition between cormorants and fishers.

The methods developed in this study were applied to data relating to commercial fishing and predation by Great Cormorants (*Phalacrocorax carbo sinensis* L. 1758) from a colony at Juodkrantė, on the western coast of the Curonian Lagoon in Lithuania. In an investigation of cormorant diets in this area, 76% of surveyed birds were observed to have consumed European perch [24]. European perch is the fourth most abundant fish species in the Curonian Lagoon [36] and one of the most important commercial species, with annual commercial landings for the combined Lithuanian and Kaliningrad (Russia) regions of the Lagoon of 100–140 tonnes live weight (Fisheries Dept., Ministry of Agriculture, Lithuania). Comparisons between the estimates of predation selectivity of cormorants and fishing selectivity of commercial gillnets offer useful insights into the degree of competition between commercial fishers and this increasingly abundant piscivorous bird.

## Materials and Methods

### Ethics statement

All animal work was conducted on public land and waterways, did not involve protected, threatened or endangered species, and complied with relevant national and international guidelines and legislation. Permits to perform fish surveys at the Curonian Lagoon, Permit No. 01 in 2009 and Permit No. 001 in 2010, were issued to the Nature Research Centre (NRC) by The Environmental Protection Agency under the Ministry of the Environment of the Republic of Lithuania. All live animals removed from sampling nets were humanely killed by spiking the head, with either a dissecting needle for smaller fish or a scalpel blade for larger specimens, to destroy brain function. Disposal of the carcasses of unpreserved specimens was in accordance with NRC Laboratory of Marine Ecology bio-security protocols. Institutional animal ethics considerations and approvals were met via the Research Council of Lithuania project application procedural requirements.

### Sampling

**Figure 1:**
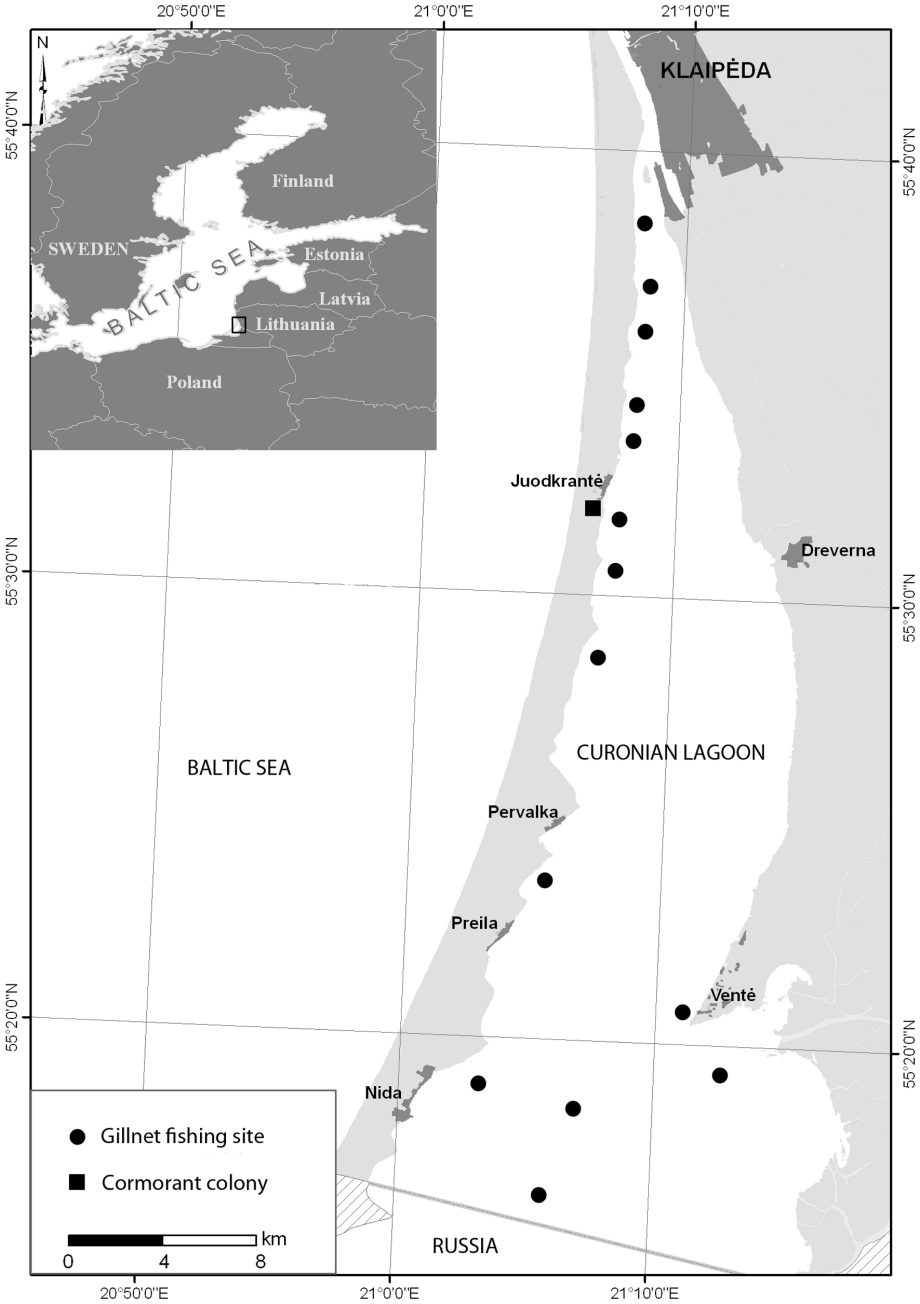
Study area, showing the Juodkrantė Great Cormorant (*Phalacrocorax carbo* L. 1758) colony (▪), and perch (*Perca fluviatilis* L. 1758) sampling sites (•).

Perch were sampled from the northern part of the Curonian Lagoon during the summer periods (July) of 2009 and 2010. The Lagoon is a 1584 km^2^ freshwater basin, located in the south-east Baltic Region and connected to the Baltic Sea through the 500 m-wide Klaipėda channel (Fig. 1). For a substantial part of the year the Lagoon is frozen, with a thick ice cover, and during autumn and spring some fish migrate outside the study area, making seasonal sampling logistically and biologically unrealistic. Sampling sites were chosen from among the deeper areas of the Lagoon, utilized as habitat by most perch populations, and to the north and south along the Nerija coast from the Juodkrantė Great Cormorant colony, as well as the southern part of the Lithuanian zone where cormorants are more active. The shallow banks and areas in the north-eastern part of the Lagoon were not sampled because they are less used by fisheries.

Samples were taken from 14 sites using a set of 11 spun nylon gillnets (mesh sizes: 14, 17, 21.5, 25, 30, 33, 38, 45, 50, 60 & 70 mm) [37], as described in Troynikov et al. (2011) [38]. A minimum size limit of 18 cm (*L*_T_) applies to the commercial fishery and the mesh size of gillnets used commercially in the Curonian Lagoon is limited to 40–45 mm (knot-to-knot).

Pellets of Great Cormorants at the Juodkrantė colony were collected for dietary analysis during the breeding season from the beginning of March to the end of July. In each instance 15–20 pellets were sampled at 10 day intervals in 2009 (n = 298) and 2010 (n = 276). Pellets were analysed according to the standard methodology described by Carss et al. (1997) [39]; for a detailed description see Pūtys and Zarankaitė (2010) [24]. Fish lengths and weights were reconstructed using allometric relationships, estimated from a reference collection. The length distribution data obtained from these pellets and the sampling nets are listed in Table 1.

**Table 1:** Observed numbers by length-class of European perch caught in gill nets of increasing mesh size (G (mm)), and by Great Cormorants (GC).

Length-class (cm)	G_14_	G_17_	G_21.5_	G_25_	G_30_	G_33_	G_38_	G_45_	GC	Total
2	0	0	0	0	0	0	0	0	1	1
3	0	0	0	0	0	0	0	0	13	13
4	0	0	0	0	0	0	0	0	71	71
5	0	0	0	0	0	0	0	0	250	250
6	0	0	0	0	0	0	0	0	244	244
7	0	0	0	0	0	0	0	0	588	588
8	0	0	0	0	0	0	0	0	952	952
9	0	0	0	0	0	0	0	0	587	587
10	5	0	0	0	0	0	0	0	251	256
11	46	5	0	0	0	0	0	0	128	179
12	30	22	0	0	0	0	0	0	93	145
13	37	54	2	2	0	0	1	0	81	177
14	12	37	6	2	1	0	0	0	82	140
15	1	19	15	0	1	1	0	0	111	148
16	5	3	28	5	0	0	0	0	79	120
17	2	4	23	31	2	0	1	1	70	134
18	0	3	16	42	1	0	0	2	39	103
19	0	3	10	35	6	1	0	1	40	96
20	1	1	9	22	13	2	1	0	28	77
21	0	3	3	16	18	7	0	1	18	66
22	0	1	4	11	11	18	0	0	14	59
23	0	2	4	5	12	10	0	0	8	41
24	0	0	0	4	7	4	4	0	6	25
25	0	1	0	4	5	7	10	1	4	32
26	0	0	1	3	8	6	20	1	4	43
27	0	0	1	2	7	1	21	1	1	34
28	0	0	2	1	3	1	14	1	2	24
29	0	0	0	1	3	0	9	4	0	17
30	0	0	0	0	1	0	4	10	0	15
31	0	0	0	0	1	0	4	9	1	15
32	0	0	0	0	0	0	1	5	0	6
33	0	0	0	0	0	0	0	2	0	2
34	0	0	0	0	0	0	0	1	0	1
35	0	0	0	0	1	0	1	0	0	2
Total	139	158	124	186	101	58	91	40	3766	4663

### Estimation procedure

This study extends the maximum likelihood estimation procedure for selectivity functions developed by Kirkwood and Walker (1986) [40]. The likelihood function is based on the assumption that for each gear type *i* and length-class *j*, the catches *n_ij_  = * f*_i_μ_j_ S_ij_* are independent observations from a Poisson distribution with mean *μ_j_ S_ij_*, where *μ_j_* is the relative proportion in the population from length-class *j, S_ij_* is a selectivity function and f*_i_* is fishing power;
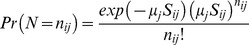
(1)


Then, with an assumption that each gear has fishing power equal to 1, at the maximum of the log-likelihood function;
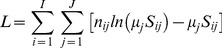
(2)


and parameter μ*_j_*  =  Σ n*_ij_*/Σ S*_ij_*, *i* =  1,..., *I* (see [40]). The selectivity function S is proportional to the conditional probability P (“*catch* ”| j) of fish to be captured given size-class *j*. Thus, the fundamental relationship between the selectivity function S*_ij_*, the size distribution in the sampled fish population *f_j_* (prior distribution) and size distribution *p_ij_* in a sample by gear type *i* (posterior distribution) can be formulated as the Bayesian theorem:
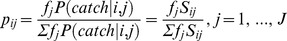
(3)


Given that fishing power may only affect sample size, but not the size distribution in a sample, values *p_ij_* are proportional to Pr (N =  n*_ij_*) in model (1) and size-frequency data can be used in estimator (2). This consideration has an important practical implication; it allows for the use of normalised size-frequency data collected by different types of fishing gears (including Great Cormorants) that may have incomparable fishing efforts and different fishing powers. The only values that need to be estimated in this case are the parameters of our nominated selectivity curves. Following Kirkwood and Walker (1986) [40], gamma probability density functions (*pdf*), rescaled to modal values of one, were used to describe fishing mesh net selectivity. Accordingly, the functional form used to model selectivities as a function of length, *l,* is:
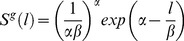
(4)where *g* denotes fishing gear.

The length at maximum selectivity for net *i* is proportional to the mesh size *m*_i_, and variance *θ_2_* is a constant over different nets (principle of geometrical similarity) so that:
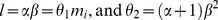
(5)


By adopting a specific functional form for the fishing gear selectivity function and solving equations (5) with respect to *α* and β, only two parameters, *θ*_1_ and *θ*_2_, need to be estimated. However, the Great Cormorant “gear” type cannot be rescaled to any mesh size and the selectivity parameters must have independent values. An investigation of the cormorant size-frequency catch data reveals a distribution with a heavy right-hand tail, indicative of a lognormal shaped selectivity function. Therefore a log-normal *pdf*, rescaled to a modal value of one, was used to describe cormorant predation selectivity. The functional form used to model cormorant selectivity as a function of length, *l,* is
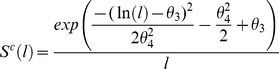
(6)


with mode at 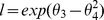 and variance;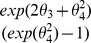; and where *c* denotes cormorants.

Thus in distinction from Kirkwood and Walker (1986) [40], there are two extra parameters to estimate. The log-likelihood function of the whole data set is:
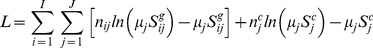
(7)


Maximum likelihood estimates for parameters of interest, *θ_1_, θ_2_, θ_3_,* and *θ_4_,* were obtained using the Nelder-Mead simplex method [41]. All statistical analyses were performed using the R software environment (Version 2.13.0, R Development Core Team, 2011). The Nelder-Mead method was employed using the general-purpose optimization function *optim*, available in R standard libraries. All source code, together with example usage scripts and the data used in this paper are available with instructions for their use at https://www.github.com/awhitten/corselect.

## Results

European perch catch numbers were converted to normalised frequency data before applying the maximum likelihood estimator (7). Initial values of the parameters of interest (a requirement for the optimization procedure) were approximated using the following heuristics: as starting points, (1) the mode of an estimated selectivity function can be set close to the value corresponding to the mode of the length-frequency distribution from any particular gear, and (2) the value of the variance can be set around two-thirds the width of that same frequency distribution. The maximum likelihood estimates of the four parameters of interest are shown in Table 2. Parameter estimates were used to determine a mode and variance for each of the fishing gears and cormorant predation selectivity functions according to the equations presented under *Estimation Procedure*. The variance for all fishing gear selectivities was 12.10. The mode and variance for cormorant predation were 8.00 cm and 10.8 respectively.

**Table 2:** Parameter and standard error estimates for theta one and two, used to describe gamma shaped selectivity curves for categories of fishing gear, and for theta three and theta four, used to describe a log-normal shaped selectivity curve for cormorant predation.

Parameter	Estimate	Standard Error
θ1	0.71	0.05
θ2	12.94	7.23
θ3	2.13	0.43
θ4	0.41	0.37

**Figure 2:**
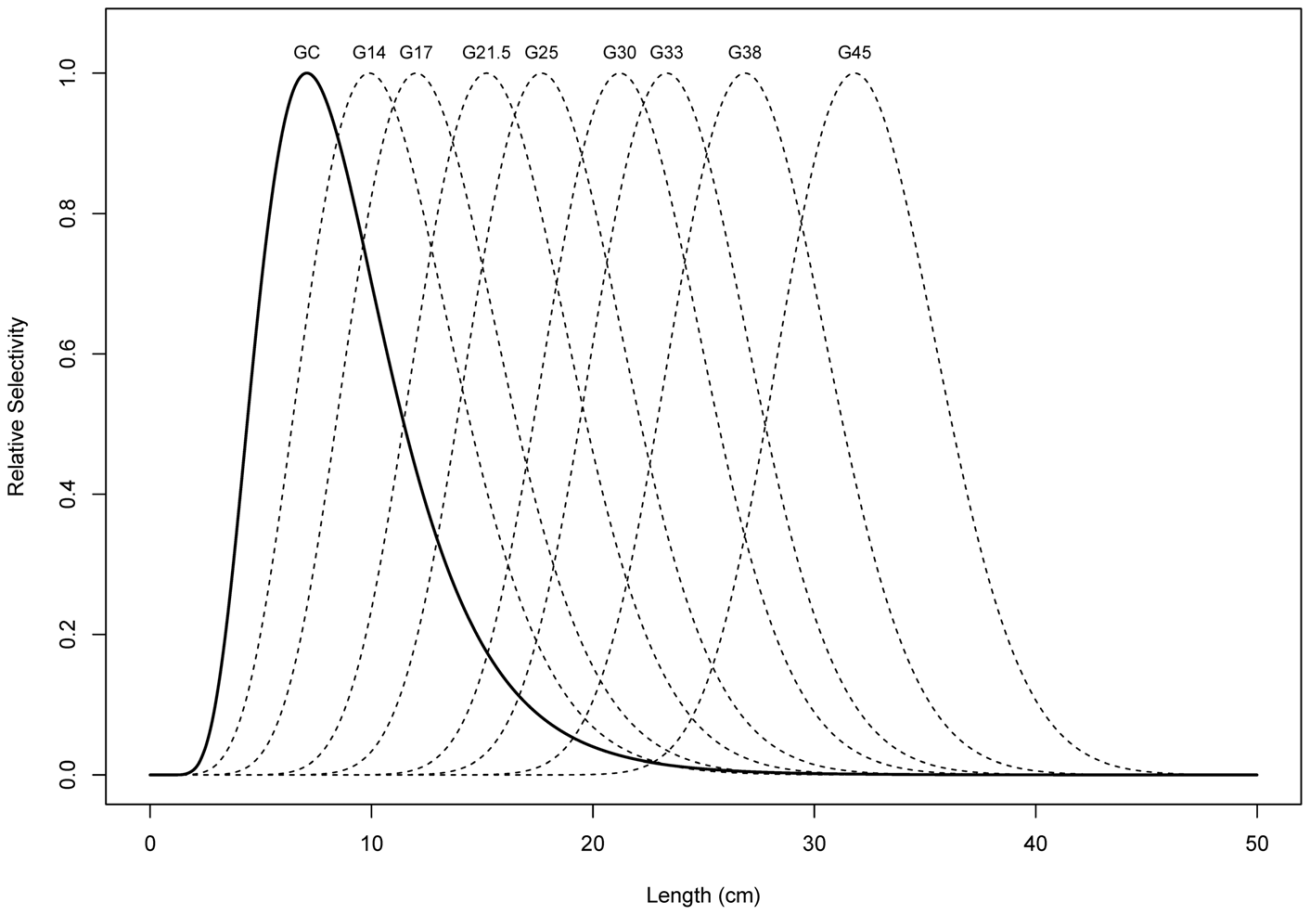
Estimated relative selectivity as a function of length of European perch for eight gillnets with increasing mesh size (dashed lines, marked “G” with mesh size in mm) and Great Cormorants (solid line, marked “GC”).

Cormorant selectivity for the perch population in the Curonian Lagoon was similar to that of the 14 mm gillnet with around 70% area of overlap. However, apart from the differences in modes (7.1 cm for Great Cormorants compared with 9.9 for the 14 mm gillnet) the selectivity estimates differed in the tails of their distributions, with the cormorants having a much steeper left tail and a slightly steeper right tail (Fig. 2). Overlap with the 38 mm gillnet was <2% and there was negligible overlap with the 45 mm commercial sized nets showing cormorants rarely consumed fish in size classes targeted by commercial fishers. Indeed, the intersection point between cormorants and 38 mm selectivity curves occurs close to the minimum legal size limit of 18 cm total length.

## Discussion

Our results show there is limited direct interaction between Great Cormorant predation and commercial fishing in the Curonian Lagoon. Only a small proportion of perch (<2% of the area of intersection between the commercial gill net and cormorant selectivity functions) selected by commercial-sized gill nets were of a size that are regularly consumed by cormorants. Although no recreational catch data were available, the very small number of legal sized perch in the cormorants' diet suggests that direct interaction with this fishing sector should also be low.

Estimated selectivity curves indicate little to no direct competition between cormorants and fishers for commercial-sized perch, but they do reveal strong selection of sub-legal sized perch by cormorants. The estimated average annual consumption rate of perch by Great Cormorants in the Lithuanian part of the Curonian Lagoon is 118 t which is 2.5 times commercial landings and represents 18% of the fish biomass that cormorants from this area consume (Ž. Pūtys unpubl. data). Our findings indicate almost all this consumption is of juvenile perch, yet to reach the commercially targeted component of the stock.

Substantial consumption of juvenile perch by cormorants might be expected to affect recruitment to the fishery and therefore catches, but landings throughout the entire Lagoon increased by 22% during the period 2007–2011. Whilst increased catches do not necessarily reflect changes in abundance, others have shown substantial fish consumption by cormorants does not lead to stock declines in prey populations [7], [12]. There may be several mechanisms responsible for such effects: We outline one scenario based on the literature. If cormorant numbers have increased in proportion to prey abundance due to a reduction in other predators [42], [43], such as a depletion of pikeperch (*Sander lucioperca* L. 1758) by fishing [44], then reductions in fish population densities by cormorants may stimulate growth and fecundity rates in perch through compensatory mechanisms related to reduced intra-specific competition in semi-closed fishing grounds, thereby improving the rate of recruitment (e.g. [45]). Under this hypothesis, as cormorants reduce their prey to more stable densities, there will be a gradual attrition in their numbers commensurate to overall prey availability [46].

This study also provides a computational method for the simultaneous estimation of fish length selectivity parameters for multiple capture methods, no matter whether they are predators or types of fishing equipment. The approach has general ecological application potential, and could be used as a starting point when comparing the specificity and relative efficiency with which piscivorous birds or other animals predate upon fish. Although several methods exist for direct estimation of selectivity parameters (e.g. [33], [40]), such studies have previously been restricted to methods that deal only with single gear categories (and thus single functional forms). The method presented here allows multiple gear types and predators to be included within a single estimation procedure, avoids the need for assumed equality in fishing power, and enables direct estimation from length composition data alone. This study thus presents a key step toward modelling and understanding the impacts of competing predators on fish stocks, without the need to extend analyses to highly complex and data-hungry integrated analysis methods. Importantly, data collection costs can be reduced with this method as it permits the use of historical data that may not otherwise have been intended for gear selectivity estimation.

The methods developed in this study cannot, in isolation, test the assertion by fishers that increased numbers of Great Cormorants in the colony at Juodkrantė are negatively affecting commercially important fish stocks in the Curonian Lagoon. However, by providing important inputs to age- or size-structured population models, these methods enable a two-step approach to the examination of population level effects of cormorant predation on European perch stocks. Age- or size-structured population models, including simulation models, could enable better estimates of total mortality on young fish and thus improve predictions of future recruitment to the fishery. Indeed, models that explicitly consider both gear selectivity and predator induced natural mortality can significantly improve stock assessment models, especially those that address ecosystem concerns [47]. Thus future research and stock assessment projects would benefit from incorporating the methods presented in this study. Such analyses would likely improve understanding of the full effects of size-selective perch predation by Great Cormorants.

Perceived versus actual effects of cormorant populations on fishable stocks can cause unnecessary concern for commercial fishers. Understanding the wide range of ecological processes at play is critical to future management of their fishery. In addition to continuing to monitor the abundance and age-structure of fish populations, concurrent surveys of cormorant numbers and their diets would permit estimates of annual perch consumption and support population modelling. Together these efforts would help determine the effect of cormorant predation on the dynamics of the fishable stock, and whether it might be important to reduce that predation in order to protect future recruitment to the fishery.

The selectivity estimation procedure in this study provides a useful link in determining the relative impacts of fishing and natural predation on fisheries, not only for the Curonian Lagoon, but for fisheries generally. Further development of this method is recommended to allow for the estimation of different selectivity curves, and for methods to determine the best selectivity functions relating to different fishing gears or predators of interest.
