# Correction: Cormorant Catch Concerns for Fishers: Estimating the Size-Selectivity of a Piscivorous Bird

**DOI:** 10.1371/annotation/b59e978e-722d-4835-9350-f2f92d6f3738

**Published:** 2014-01-02

**Authors:** Vladimir Troynikov, Athol Whitten, Harry Gorfine, Žilvinas Pūtys, Eglė Jakubavičiūtė, Linas Ložys, Justas Dainys

A mathematical expression of "variance" in the first line immediately below Equation number 6 in the "Estimation procedure" of the Materials and Methods is incorrectly given. The correct expression is: *exp*(2*ϴ*_3_+*ϴ* ^2^_4_)(*exp*(*ϴ* ^2^_4_)-1). 

